# Application of Human Posture Recognition Based on the Convolutional Neural Network in Physical Training Guidance

**DOI:** 10.1155/2022/5277157

**Published:** 2022-06-28

**Authors:** Qingyu Wang

**Affiliations:** College of Sport, Xuchang University, Xuchang 461000, Henan, China

## Abstract

The application of sports game video analysis in athlete training and competition analysis feedback has attracted extensive attention, but the traditional sports human body posture estimation method has a large error between the athlete's human body posture estimation results and the actual results in the complex environment and the athlete's body parts are blocked. Therefore, this study proposes a convolutional neural network for athlete pose estimation in sports game video. Based on the improved model, multiscale model, and large perception model, a superimposed hourglass network is constructed, and the gradient disappearance problem of the convolutional neural network is solved using intermediate supervision. The experimental results show that the athlete pose estimation model based on the convolutional neural network can improve the accuracy of athlete pose estimation and reduce the negative impact of occlusion environment on athlete pose estimation to a certain extent. In addition, compared with other athletes' standing posture estimation methods, the model has competitive advantages and high accuracy under widely used standard conditions.

## 1. Introduction

With the continuous development of computer vision technology, sports video analysis technology has been widely used in the event analysis of sports competitions. It can provide athletes and coaches with corresponding data as a reference through video analysis and make a relatively systematic evaluation of individual athletes' and groups' performance in sports competitions [1]. In recent years, the number of sports videos has increased geometrically, and at the same time, there is a large amount of interference information in the huge amount of sports videos [2]. The information that athletes and coaches need and pay attention to occupies relatively little in the game video, so how to quickly find the needed information in the massive video information has become a research hotspot in sports video technology.

Sports competition training analysis is mainly divided into technical analysis and tactical analysis, in which technical analysis is the analysis of athletes' personal technical ability, movement standardization, and physical quality in the competition, and tactical analysis is the guidance of the competition and the application of tactical methods, etc. [3, 4]. Through the analysis of sports competition videos, it can effectively help athletes understand their competitive level and competitive strength of competitors and learn the competitive technology of excellent athletes [5]. With the continuous application of human posture estimation and human motion recognition technology based on the deep neural network, sports training analysis technology based on human posture estimation has also developed rapidly. The athlete's human posture recognition and estimation technology can recognize and estimate the athlete's posture in the high-resolution recorded competition video, decompose the athlete's posture in multiple dimensions, obtain the real action data of the athlete in the competition, and then combine the analysis algorithm with scientific analysis to obtain the final evaluation result of the athlete's performance [6, 7]. In addition, the same movement of athletes in the same sport can be compared horizontally to find out the gap and problems between athletes' movements and standard movements, which will help athletes improve and reduce the possibility of repeated training [8]. At the same time, it allows athletes to more intuitively understand the training content and give rapid feedback to reduce sports injuries caused by irregular sports [9]. However, the traditional athlete posture recognition and estimation methods cannot fully extract image features. Although the time required is relatively small, when the athlete's posture changes greatly in training, the error between the estimation results and the actual results will become larger [10]. Therefore, estimating the posture recognition of skilled athletes in complex environment has become a challenge for sports video analysis technology.

Combined with the convolution neural network, this study studies the method of athlete posture estimation in sports training. This study creatively constructs a layered hourglass network based on improved module, multiscale module, and large perception field module. The intermediate supervision method is used to avoid the gradient vanishing problem of the convolutional neural network. Compared with the traditional athlete posture estimation method in image feature information extraction, this method has more practical application value. It solves the problem that there is a large error between the estimated results and the actual results when the environment in the sports video is complex or the athletes' body parts are blocked.

To solve the above problems, this study proposes a convolutional neural network research on athletes' human posture estimation technology in sports game training, which is mainly divided into three parts. The first part introduces the development of human posture estimation technology. The second part is the construction of the athlete posture estimation model based on the convolutional neural network and introduces the adopted model. In the third part, the performance experiment and simulation experiment of the athletes' human posture estimation model based on the convolutional neural network are carried out, and the experimental results are analyzed.

## 2. Related Work

Since the 1980s and 1990s, people have proposed and studied the corresponding human posture estimation methods. However, in the early research stage, computer equipment and deep learning technology are still in the exploration and development stage. Therefore, most of the traditional human posture estimation methods are based on the graphical structure model [11]. Some scholars have improved the graph structure based on statistical combination to obtain the tree structure, which can reduce the computational complexity, but it is difficult to correctly estimate the human posture in the case of complex environment scene or partial occlusion of the human body [12, 13]. To solve these problems, it is suggested to add corresponding constraints to the occluded part of the human body to achieve improvement and combine the confidence propagation algorithm to estimate the corresponding human posture. Therefore, even in the case of human occlusion, human joint points can be predicted relatively accurately [14]. With the in-depth study of human posture and the development of learning technology, many algorithms have achieved good results in the field of human posture estimation.

The current human pose estimation is mainly divided into overall human pose estimation algorithms and human pose component estimation algorithms [15]. The algorithms for overall human pose estimation usually use a nonlinear mapping between images and nodal positions to achieve human pose estimation [16, 17]. A deep neural network-based human pose estimation algorithm has been proposed to obtain pose estimation values with high accuracy using DNN regression quantities, which has the advantage over other algorithms in that it performs pose inference through a holistic approach [18]. It has also been proposed to incorporate domain prior knowledge in the DCNN framework and combine the variability mixture component for end-to-end human pose estimation [19]. Normalized distances in overall human pose estimation methods like this can have a large impact on the localization of nodes. For the human component estimation algorithm, the human model is built based on the appearance and position of the component or joint, and then, the corresponding optimization is performed to obtain the corresponding energy function [20]. Traditional joint point models are manually performed with image feature design, which makes the real appearance of their representations have a large error, while deep learning can solve this problem. It has been proposed to combine human joints, parts affinity force fields combined with greedy algorithms for learning body parts and individual connections in images through nonparametric representations, and such an approach can achieve real-time performance while maintaining low error regardless of the number of individuals in the image [21, 22]. However, body pose estimation by body parts is prone to loss of body information, while the bottom-up approach increases the chances of joint point misdetection. Therefore, in the field of sports game training and analysis, there are many problems in human posture estimation technology, which need to be further developed and solved.

## 3. Construction of the Athlete Posture Estimation Model Based on the Convolutional Neural Network

The convolutional neural network has two characteristics: local sensing and parameter sharing. Local sensing, namely, the convolution neural network, proposes that each neuron does not need to perceive all pixels in the image. Only the local pixels of the image are sensed, and then, the local information is combined at a higher level to obtain all the representation information of the image. The nerve units of different layers are connected locally; that is, the nerve units of each layer are only connected with some nerve units of the previous layer. Each nerve unit only responds to the areas within the receptive field and does not care about the areas outside the receptive field. Such local connection mode ensures that the learned spatial local mode of convolution check input has the strongest response. Due to the complex background of the application scene, the flexibility of athletes' limbs, and the serious occlusion of various parts of the human body, the traditional human posture estimation methods show limited representation performance in practical application and produce unsatisfactory posture estimation results. The athletes' attitude estimation method based on the convolutional neural network has good nonlinear transformation ability, and the improvement of the convolutional neural network and other auxiliary methods can further improve the athletes' attitude estimation accuracy in game training.

### 3.1. Multiresidual Modular Convolutional Neural Network Athlete Pose Evaluation Model

**Figure 1:**
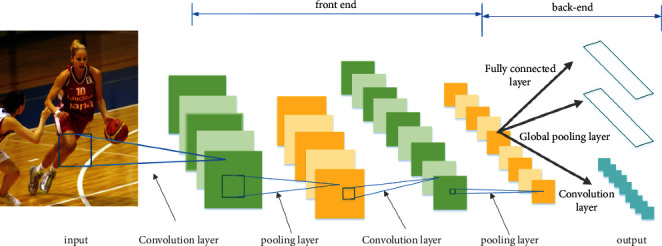
Simplified architecture of the convolutional neural network.

Among the deep learning models of human pose estimation algorithms, convolutional neural networks have the unique advantage of being feedforward neural networks with deep structure and after continuous development currently contain mainly convolutional layers, pooling layers, fully connected layers, activation functions, normalization layers, inputs, outputs, etc. The pool layer is sandwiched in the middle of the continuous convolution layer to compress the amount of data and parameters and reduce overfitting. If the input is an image, the main function of the pooling layer is to compress the image [23]. A simplified architecture of the convolutional neural network is shown in Figure 1.

In convolutional neural networks, the convolutional layer is a feature extraction layer by having convolutional kernels for abstract feature extraction, to reduce the number of parameters; it mainly takes local sensing and weight sharing methods [24]. The pooling layer, on the other hand, reduces the number of neurons by counting and downsampling the features [25]. The following equation is the convolution operation.

(1) $$\begin{array}{c} F\left(i,j\right)=X{\left(3{}^{*}3\right)}_{i\times j}⊗f\left(3{}^{*}3\right), \end{array}$$

where the *F*(·) denotes the features after convolution, (*i*, *j*) denotes the pixel points of the image, *X*(·) denotes the matrix of the pixel points and the neighboring pixel points at the corresponding position, and *f*(·) denotes the filter. If the size of the input image is denoted as *H* × *W* and the size of the convolution kernel is *N*, the size of the feature map obtained after the operation with the convolution step is shown in *S* as follows:

(2) $$\begin{array}{c} H_{\text{feat}}=\frac{H-N}{S}+1, \end{array}$$

(3) $$\begin{array}{c} W_{\text{feat}}=\frac{W-N}{S}+1. \end{array}$$

If the complementary zero operation is added, the input image size is *M*, as shown in equation:

(4) $$\begin{array}{c} W_{\text{feat}}=\frac{M+2K-N}{S}+1. \end{array}$$

Although the performance of the convolutional neural network in athlete posture estimation is better than that of traditional methods, there are still some problems, such as gradient problem and small sensing domain of the feedforward network. Therefore, this study selects a convolutional neural network with stacked hourglass structure and improves it combined with multiple modules [26]. The superimposed hourglass grid is constructed using grid and intermediate supervision method, which not only solves the problem of gradient disappearance, but also has high network performance and athlete attitude estimation accuracy. However, the performance of a single module cannot meet the requirements of athletes' posture estimation technology. Therefore, this study selects large sensory field module, improved module, and multiscale module for network construction.

**Figure 2:**
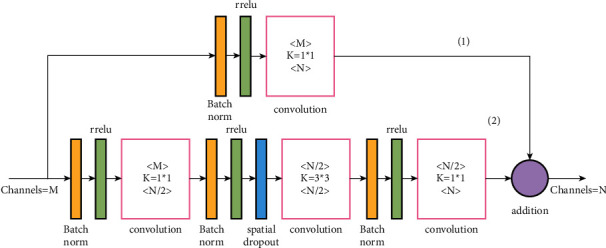
Improved hourglass grid stacking diagram.

The accurate performance of the neural network is improved to estimate athletes' posture, so that the neural network can carry out very in-depth training. However, its own unit mapping will also make the response change increase with the increase in depth. Therefore, the laminated hourglass network will have a great response impact when training deeper networks, which increases the difficulty of parameter optimization and estimation error. Based on the traditional laminated hourglass grid, the improved laminated hourglass grid replaces the unit mapping with normalization layer, ReLU, and convolution and introduces spatial attenuation layer 3*∗*3 before the convolution, as shown in Figure 2.

When estimating athletes' posture in game training, the correlation and connection between the whole body parts and joints are affected by the effective receptive field of neurons to the surrounding area. The size of the mapping range of the pixel points corresponding to each layer of the output feature mapping in the convolution neural network on the original image is the receptive field, which corresponds to the receptive field of the first layer, as shown in the following equation:

(5) $$\begin{array}{c} F_{i}=F_{i-1}+\left(k-1\right)*\text{stride}\left(i\right), \end{array}$$

where is the *F*_*i*−1_ perceptual field size of the previous layer, *k* denotes the current convolution kernel size, and stride(*i*) denotes the step product of the previous (*i* − 1) layers; i.e., stride(*i*)=stride(1)*∗*stride(2)*∗*....stride(*i* − 1).

**Figure 3:**
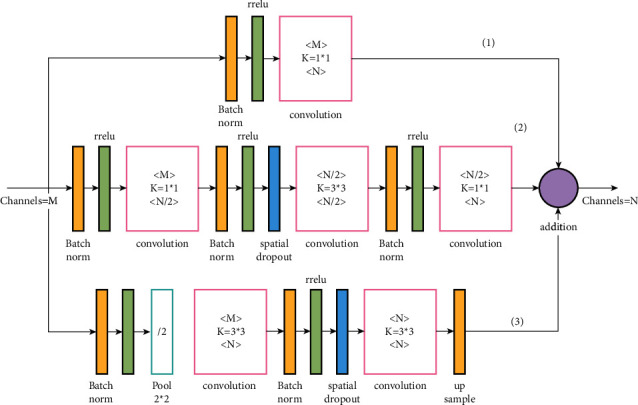
Schematic diagram of large receptive field residual module.

The effective receptive field of the improved module is still very small, so a large receptive module is constructed based on the improved module. The schematic diagram of large magnetic field residual module is shown in Figure 3.

When helping the deep residual learning to achieve a breakthrough in the task of image recognition and classification, the module is shown in the following equation:

(6) $$\begin{array}{c} p_{i+1}=h\left(p_{i}\right)+F\left(p_{i},W_{i}^{F}\right). \end{array}$$

The corresponding input of the first *i* residual module is denoted as *p*_*i*_, and the output is denoted as *p*_*i*+1_, *F* denotes the convolution that forms the stack, and *h*(*p*_*i*_)=*p*_*i*_ denotes the unit mapping. The large sensory field residual module is shown in the following equation:

(7) $$\begin{array}{c} p_{i+1}=h\left(p_{i}\right)+F\left(p_{i},W_{i}^{F}\right)+Q\left(p_{i},W_{i}^{Q}\right). \end{array}$$

**Figure 4:**
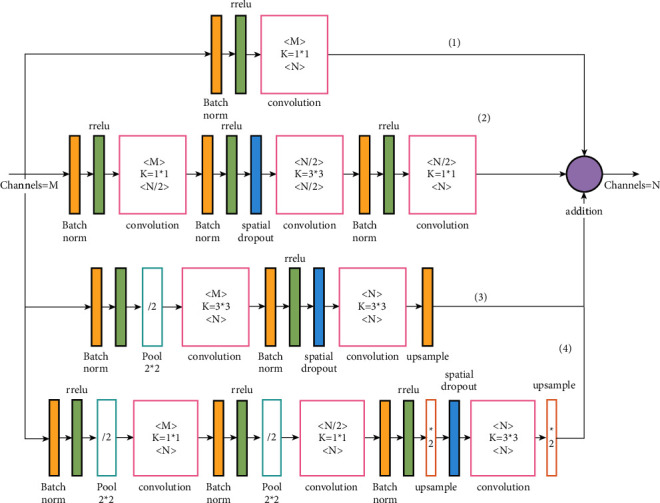
Schematic diagram of multiscale residual module.

The offset problem is one of the common problems faced by equipment manufacturers who use stepping or servo motors in the process of equipment setting, commissioning, and use. The deviation is caused by improper mechanical fittings, mutual interference of equipment in the workshop, or improper grounding wire treatment in the equipment setting. The problem of large positioning error of joint points is caused by the change in athletes' body shape in the competition video. This requires the integration of multiscale modules on the basis of large receiving field modules. This requires the integration of multiscale modules on the basis of large receiving field modules, as shown in Figure 4.

As can be seen from the figure, the multiscale module in (1), (2), and (3) is the same as the large sensory field module, while branch (4) *Q*(*p*_*i*_, *W*_*i*_^*Q*^) is a new addition with the structure of pool2*∗*2+conv3*∗*3+pool2*∗*2+conv3*∗*3+upsample2*∗*2+upsample2*∗*2, from which we can see that there are two downsamples in this branch, which means that the feature map size is 25% of the input feature map, and after upsampling to the input feature mapping size, it is fused with the other three branches in series. In the other branches, the feature map size of branch (3) is 50% of the input feature map, and the feature map sizes of branches (1) and (2) remain unchanged. The expressions are shown in the following equation:

(8) $$\begin{array}{c} p_{i+1}=h\left(p_{i}\right)+F\left(p_{i},W_{i}^{F}\right)+Q\left(p_{i},W_{i}^{Q}\right)+G\left(p_{i},W_{i}^{G}\right). \end{array}$$

**Figure 5:**
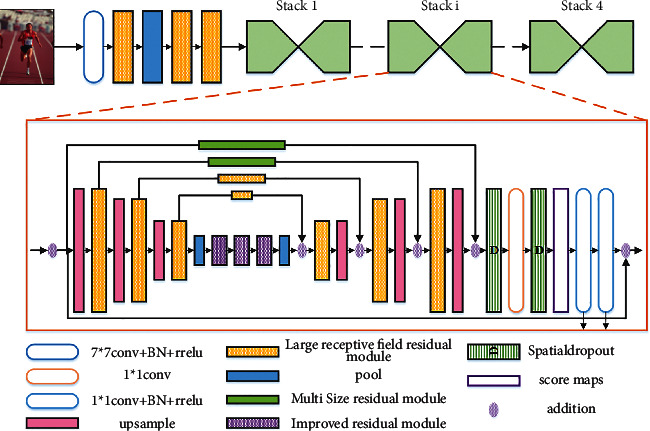
Overall framework of the convolutional neural network.

Figure 5 shows the overall framework of the convolutional neural network. As shown in the figure, it can be divided into two parts, the first half and the second half. The first half of the framework is composed of convolution, large field of view module, and pool, which is used to learn athletes' pose estimation in the feature map. After convolution kernel operation, the size of the feature map remains in the second half. The second half of the frame is composed of hourglass subgrid structure, with intermediate supervision at the end of each hourglass, and the feature map is processed multiple times between high resolution and first resolution to form a stacked hourglass grid.

### 3.2. Off-Node Prediction and Athlete Pose Estimation Dataset

The predicted positions of the joint nodes are mainly regressed by the score map, i.e., the score map corresponding to each hourglass subnetwork and human joint point in the multiresidual convolutional neural network. Let the actual labeled position of the joint point be denoted as *V*={*V*_*k*_}_*k*=1_^*K*^ and position of the first *k* joint point is denoted as *V*_*k*_=(*x*_*k*_, *y*_*k*_), and then, the fractional map of the actual position is calculated as shown as follows:

(9) $$\begin{array}{c} S_{k}\left(t\right)∼N\left(v_{k},\sum \right), \end{array}$$

where the predicted positions of the nodes are denoted as *t* ∈ *R*^2^, and ∑ denotes the unit matrix. If the number of nodes is *K* one, the number of predicted score maps obtained for each hourglass subnetwork is *K*, i.e., $\overset{\wedge }{S}={\left\{{\overset{\wedge }{S}}_{k}\right\}}_{k=1}^{K}$. The hourglass end loss function is shown in the following equation:

(10) $$\begin{array}{c} L={\sum_{m=1}^{M}\sum_{k=1}^{K}\left\|S_{k}-{\overset{\wedge }{S}}_{k}\right\|}_{2}^{2}, \end{array}$$

where *M* denotes the number of samples. The position at the highest score on the score graph of the last hourglass subnetwork after the prediction process is marked as the prediction node position, which is denoted as $\overset{\wedge }{v_{k}}$, as shown in the following equation:

(11) $$\begin{array}{c} \overset{\wedge }{v_{k}}=\underset{t}{\arg\max}{\overset{\wedge }{S}}_{k}\left(t\right),\;k=1,2,\ldots ,K. \end{array}$$

The MPII dataset, LSP dataset, and MS COCO dataset are commonly used for experimental training of athlete's pose estimation in competition video, and the LSP dataset and its extensions are included in this study. In the LSP dataset, there are many pictures of athletes with rich posture and recognized high difficulty in posture estimation, and the number of joints for human posture estimation is 14 joints, which is challenging for the experiment. The estimated number of joints in human posture is sixteen joints, and the evaluation criterion is the percentage of correct joints, i.e., PCK; after normalizing the data annotation, if the joints are still within the distance of the threshold value, then the joints are correct, as shown in the following equations:

(12) $$\begin{array}{c} D_{p}=\frac{\sqrt{{\left(x^{p}-x^{g}\right)}^{2}+{\left(y^{p}-y^{g}\right)}^{2}}}{\text{HoH}}, \end{array}$$

(13) $$\begin{array}{c} \text{PCKh}@s=\frac{\sum_{p}\delta \left(D_{p}<s\right)}{\sum_{p}1}, \end{array}$$

where *s* is the threshold value and *s* ∈ {0.1, 0.2, 0.3, 0.4, 0.5}, *p* is the number of nodes, and (*x*^*p*^ − *x*^*g*^) and (*y*^*p*^ − *y*^*g*^) are the predicted and actual values, respectively. The prediction is wrong when *D*_*p*_ > and correct when the opposite is true, *PCKh*@*s*.

The number of estimated joints of human posture in the MS COCO dataset is 17, which is evaluated in terms of the average accuracy, i.e., mAP, as shown in the following equations:

(14) $$\begin{array}{c} \text{mAP}=\text{mean}\left\{AP@\left(0.5:0.005:0.95\right)\right\}, \end{array}$$

(15) $$\begin{array}{c} AP@s=\frac{\sum_{p}\delta \left({\text{OKS}}_{p}>s\right)}{\sum_{p}1}. \end{array}$$

**Figure 6:**
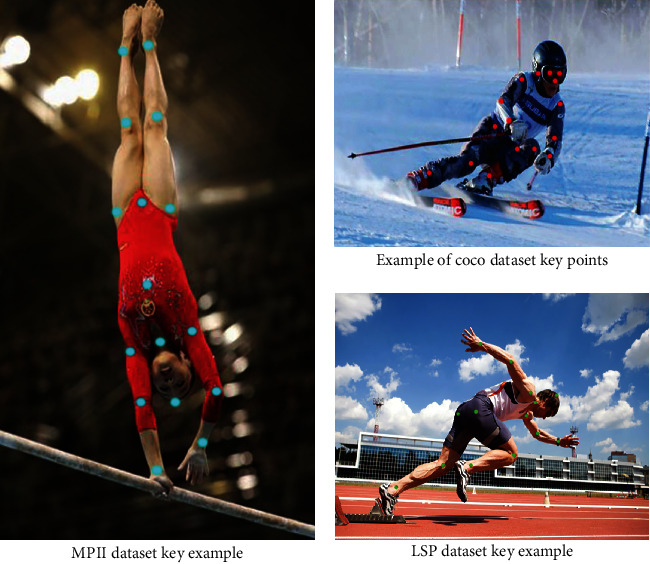
Examples of joint points in three datasets.

The threshold value of *OKSs* ∈ {0,1[], which is the evaluation criterion for the similarity between the actual and predicted values of the nodes. An example of three dataset nodes is shown in Figure 6.

## 4. Experimental Results of Athlete Pose Estimation in Sports Game Video Combined with the Convolutional Neural Network

### 4.1. Experimental Results of Performance of the Athlete Posture Estimation Model Based on the Convolutional Neural Network

**Figure 7:**
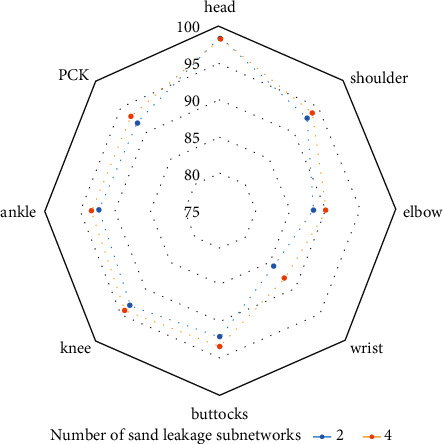
PCK results on the LSP dataset.

**Figure 8:**
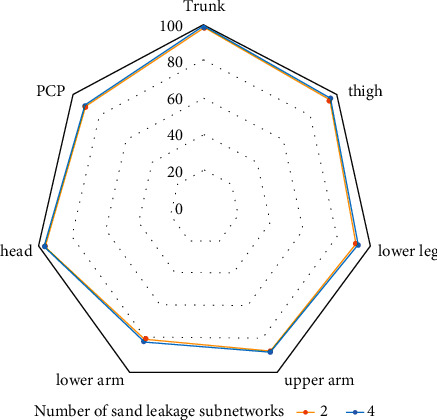
PCP results on the LSP dataset.

**Figure 9:**
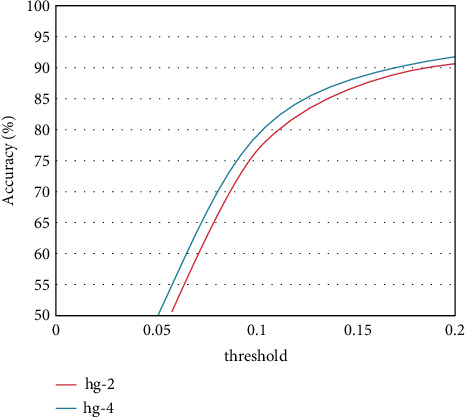
Overall PCK curve.

**Figure 10:**
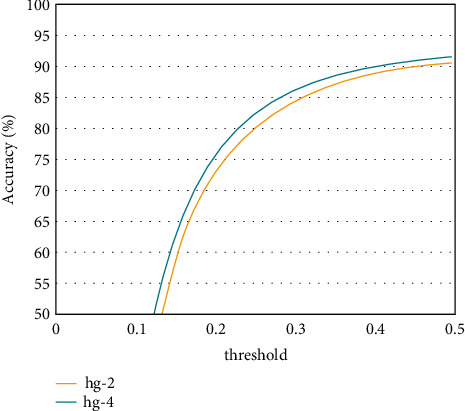
Overall PCP curve.

The convolutional neural network human posture estimation model is mainly composed of the sand leak subnetwork. Therefore, this study makes an experimental study on the impact of the establishment of sand leak subnetwork on the performance of athletes' human posture estimation. Under the same other experimental conditions, experiments are carried out on different numbers of sand leak subnetworks on the dataset. As shown in Figures 7 and 8, the experimental results of the training model composed of different number of sand leakage subnetworks under the evaluation standard conditions are shown. Figures 9 and 10 show the overall PCK and PCP curves, respectively. The horizontal coordinate represents the threshold of measurement, and the vertical coordinate represents the accuracy of each joint or different part of the athlete in the video.

From the results in the figure, we can know that the performance of the athlete pose estimation model based on the multiresidual module convolutional neural network effectively improves with the increase in the number of hourglass subnetworks. This is mainly because the individual hourglass subnetworks can only learn a limited number of image features and their effective perceptual field is relatively small, but the stacked hourglass networks are much more capable of learning feature information and can acquire more feature information. After related experiments, the number of hourglass subnetworks finally selected in this study is 4, which has the best performance in the experiment and has a certain degree of positive impact on the accuracy of the athlete's pose estimation.

### 4.2. Experimental Results of the Athlete Posture Evaluation Model Based on the Convolutional Neural Network

**Figure 11:**
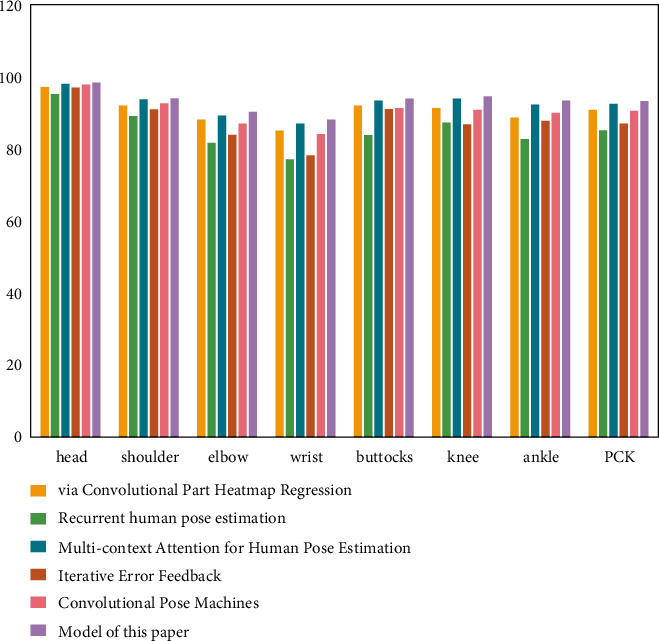
PCK standard comparison results of different athlete posture evaluation models on the LSP dataset.

**Figure 12:**
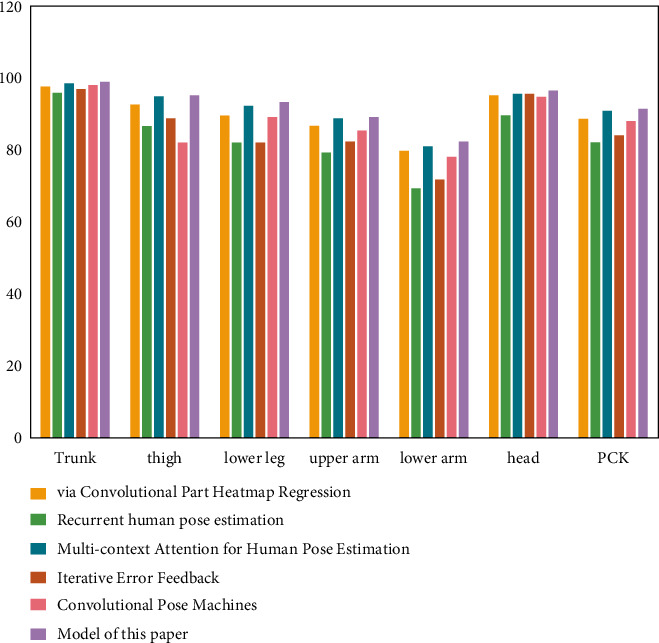
PCP standard comparison results of different athlete posture evaluation models on the LSP dataset.

To help the athletes' posture estimation model based on the convolutional neural network learn better, this study combines the LSP dataset and MPII dataset for model training. Through the performance test, the experimental comparison results of PCK and PCP are obtained, while the results of other competition video athlete posture estimation methods are from the corresponding references. As shown in Figure 11, the PCK standard comparison results of the athlete posture estimation model based on the multimodule convolutional neural network on the LSP dataset are shown.  Figure 12 shows the comparison results of PCP standard of athlete posture estimation model based on the convolutional neural network on the LSP dataset.

It can be seen from the results in the figure that the athlete posture estimation model based on the convolutional neural network is competitive with other athlete posture estimation methods. Its experimental results on LSP data show that the prediction accuracy of posture estimation is high. In the standard of athletes' posture estimation results, the index to measure and evaluate the estimation accuracy of athletes' body parts is PCP. In sports training, the upper and lower arms of athletes are most affected in the fuzzy environment. However, the data results in the figure show that the accuracy of the upper arm and lower arm of the athlete posture evaluation model based on the multiresidual module convolution neural network is 88.7% and 82.3%, respectively, both of which are relatively accurate. It can be seen that the athlete posture estimation model based on the convolutional neural network can reduce the negative impact of occlusion on athlete posture estimation to a certain extent.

As given in Table 1, after training on the MPII dataset, the results of the athlete posture evaluation model based on the convolutional neural network are compared with other widely used human posture estimation methods.

It can be seen from the table that the simple superimposed hourglass model and the improved superimposed hourglass model used for athlete posture evaluation are slightly higher than the convolution neural network based on multimodule. However, in the network design of laminated hourglass, either the simple laminated hourglass model or the improved laminated hourglass model selects 8 hourglass subnetworks, while the athlete posture evaluation model based on the multimode convolution neural network only uses 4 hourglass subnetworks. This shows that the athlete posture evaluation model based on the convolutional neural network has a certain degree of improvement in the accuracy of human posture estimation. This is mainly because the athlete posture evaluation model based on the multimode convolution neural network fully considers the influence of human body part size and model large perception field when reasoning athletes' human joints. Thus, in the model training, we can fully learn the image feature information of different sizes, and the extended perception field provides a good basis for learning the correlation of human joints. Therefore, the accuracy of athlete pose estimation in sports game video can be improved.

With the continuous diffusion of sports training data, it becomes more and more difficult for coaches and athletes to find relevant data in a large number of videos. Sports training analysis technology has been widely used in sports and sports competitions. It can not only improve the viewing experience of the audience but also systematically evaluate the performance of individual and team athletes in high-resolution video. We can also analyze athletes' postures in visual training from multiple dimensions to obtain the real movement data of athletes in the competition. These data can help coaches and athletes understand the gap between their own movements and standard movements and can also understand the economic level of other athletes through parallel comparison, to reduce the possibility of repeated training.

## 5. Conclusion

Combined with the convolution neural network, this study studies the method of athlete posture estimation in sports training. In this study, a layered hourglass network based on improved module, multiscale module, and large perceptual field module is constructed. The intermediate supervision method is used to avoid the gradient vanishing problem of the convolutional neural network. The experimental results show that the athlete posture evaluation model based on the multimode convolution neural network can effectively realize the learning of multiscale image feature information in the training process. To a certain extent, it reduces the negative impact of occlusion environment on athletes' posture estimation and improves the accuracy of athletes' joint position prediction. Compared with other athletes' pose estimation methods, the athletes' pose estimation model based on the dataset convolution neural network has certain advantages and competitiveness and shows high accuracy under the widely used standard conditions. However, this method only estimates the pose of a single athlete in the sports game video and cannot solve the pose estimation problem of multiple athletes. It has shortcomings in practical application and needs further research.

## Figures and Tables

**Table 1:** PCKh comparison results on the MPII dataset.

Method	Head	Shoulder	Elbow	Wrist	Buttock	Knee	Ankle	PCKh
Iterative error feedback	95.5	91.5	81.6	72.3	82.6	73.0	66.3	79.5
Simple stacked hourglass network model	98.1	96.0	91.1	86.9	90.1	87.2	83.4	90.1
Network model based on improved stacked hourglass	98.3	96.2	91.7	88.0	90.5	87.8	84.9	90.7
Joint subset partition and labeling	94.0	90.0	83.3	77.2	82.5	75.6	68.5	81.6
Convolutional pose machines	97.6	94.9	88.5	83.8	88.1	82.5	79.1	87.8
Model of this study	97.7	96.0	90.6	86.0	89.7	86.3	82.6	89.7

## Data Availability

The data used to support the findings of this study are included within the article.
